# Bcl-2 inhibitor resistance in diffuse large b-cell lymphoma: establishing a prognostic signature and targeting alpha protein kinase 1

**DOI:** 10.3389/fonc.2026.1729158

**Published:** 2026-01-28

**Authors:** Jingjing Ma, Yifan Wang, Hong Liu, Yuan Deng, Yuye Shi, Lulu Wei, Qiuni Chen, Chunling Wang, Liang Yu

**Affiliations:** 1 Department of Hematology, The Affiliated Huaian No.1 People’s Hospital of Nanjing Medical University, Huai’an, China; 2 Key Laboratory of Autoimmune Disease of Huaian Government, Huai’an, China; 3 Department of Pathology, The Affiliated Huai’an No.1 People’s Hospital of Nanjing Medical University, Huai’an, China; 4 Department of Hematology, The Huai’an Clinical College of Xuzhou Medical University, Huai’an, China

**Keywords:** ALPK1, diffuse large B-cell lymphoma, drug resistance, prognosis, venetoclax

## Abstract

**Background:**

Drug resistance in diffuse large B-cell lymphoma (DLBCL) contributes to poor prognosis in 30–40% of newly diagnosed patients in the era of first-line rituximab combined with cyclophosphamide, doxorubicin, vincristine, and prednisone therapy. Targeting Bcl-2 has been shown to target for improve the prognosis of these patients, based on the clinical trials of its inhibitor, venetoclax. However, venetoclax resistance in DLBCL remains a challenge. Methods: The ‘WGCNA’ package was used to comprehensively screen for Bcl-2 inhibitor-resistant genes (Bcl-2RGs) and the Bcl-2RGs signature was established using LASSO regression analysis with ten-fold cross-validation. Results: The Bcl-2 signature is an effective prognostic prediction model using Gene Expression Omnibus data analysis. In addition, we demonstrated that the inhibition of alpha protein kinase 1 (ALPK1) decreased the proliferation of DLBCL cells and increased apoptosis. ALPK1 inhibitor treatment synergized with venetoclax to suppress the ALPK1/NFκB signaling pathway. Conclusion: Overall, we showed that the Bcl-2 signature could predict the prognosis of patients with DLBCL, and that ALPK1 could be a promising target for sensitizing patients with DLBCL to venetoclax therapy.

**Methods:**

The ‘WGCNA’ package was used to comprehensively screen for Bcl-2 inhibitor-resistant genes (Bcl-2RGs) and the Bcl-2RGs signature was established using LASSO regression analysis with ten-fold cross-validation. Results: The Bcl-2 signature is an effective prognostic prediction model using Gene Expression Omnibus data analysis. In addition, we demonstrated that the inhibition of alpha protein kinase 1 (ALPK1) decreased the proliferation of DLBCL cells and increased apoptosis. ALPK1 inhibitor treatment synergized with venetoclax to suppress the ALPK1/NFκB signaling pathway. Conclusion: Overall, we showed that the Bcl-2 signature could predict the prognosis of patients with DLBCL, and that ALPK1 could be a promising target for sensitizing patients with DLBCL to venetoclax therapy.

**Results:**

The Bcl-2 signature is an effective prognostic prediction model using Gene Expression Omnibus data analysis. In addition, we demonstrated that the inhibition of alpha protein kinase 1 (ALPK1) decreased the proliferation of DLBCL cells and increased apoptosis. ALPK1 inhibitor treatment synergized with venetoclax to suppress the ALPK1/NFκB signaling pathway. Conclusion: Overall, we showed that the Bcl-2 signature could predict the prognosis of patients with DLBCL, and that ALPK1 could be a promising target for sensitizing patients with DLBCL to venetoclax therapy.

**Conclusion:**

Overall, we showed that the Bcl-2 signature could predict the prognosis of patients with DLBCL, and that ALPK1 could be a promising target for sensitizing patients with DLBCL to venetoclax therapy.

## 1 Introduction

Diffuse large B-cell lymphoma (DLBCL) is the most aggressive non-Hodgkin lymphoma with a high degree of heterogeneity (1). Approximately 30–40% of patients with newly diagnosed DLBCL experience drug resistance and relapse under a first-line rituximab combined with cyclophosphamide, doxorubicin, vincristine, and prednisone (R-CHOP) immunochemotherapy regimen. Targeted therapies for genetic abnormalities have been developed based on the definition and application of DLBCL genetic subtypes using cluster analysis. To date, several drugs targeting BTK, PI3K, JAK, IRF4, EZH2, and BCL2 have been used to improve patient outcomes (2).

Venetoclax, a Bcl-2 inhibitor, induces apoptosis by inhibiting Bcl-2 family genes (3). Venetoclax was approved by the U.S. Food and Drug Administration (FDA) for the treatment of acute myeloid leukemia (AML) (4) and more recently, has been applied to chronic lymphocytic leukemia (CLL) leading to improved treatment outcomes (5). Several ongoing and completed clinical trials of DLBCL have shown that venetoclax is a promising drug for improving DLBCL patient outcomes (6, 7). Recently, drug resistance has been observed in the clinical application of venetoclax (5). Drug resistance is a complex developmental phenomenon characterized by abnormal gene expression. Therefore, screening for key drug resistance genes may identify new therapeutic targets to reverse drug resistance and improve prognosis.

Alpha protein kinase 1(ALPK1) is a well-known innate immune sensor (8) that promotes nuclear factor kappa B (NFκB) expression (9). As a key gene in the B-cell receptor (BCR) signaling pathway, NFκB is involved in the development of DLBCL. However, the role of the ALPK1/NFκB axis in DLBCL has not been reported. In this study, a Bcl-2 inhibitor resistance gene signature was identified as a prognostic model for patients with DLBCL, and ALPK1 was found to be a target for reversing venetoclax resistance using weighted gene co-expression network analysis (WGCNA) and least absolute shrinkage and selection operator (LASSO) regression.

## 2 Materials and methods

### 2.1 Data collection and identification of differentially expressed Bcl-2 inhibitor-resistant genes

Gene expression data and the corresponding clinical data were obtained from the Gene Expression Omnibus (GEO; https://www.ncbi.nlm.nih.gov/geo/). To identify the key genes involved in venetoclax resistance, GSE223598 data (from OCI-Ly1 cells) and GSE252306 data (from SU-DHL-16 cells) were downloaded to analyze the differentially expressed genes (DEGs) in venetoclax-resistant cells compared to parental, and DEG threshold was set as: |log2-fold change (FC)|>2 and an adjusted P-value<0.05. A Venn diagram was generated showing 214 genes co-regulated in the two datasets. Among the 214 genes, 175 recorded in the GSE10846 dataset were selected as candidate genes for the prognostic models. There were 412 cases with complete gene expression and prognostic information in the GSE10846(training cohort), 470 in the GSE31312, and 221 in the GES87371 (validation cohort) datasets. The characteristics of patients in the GEO datasets have been described previously (10).

### 2.2 Establishment and validation of the Bcl-2RG prognostic signature

Gene expression data normalized to RMA in GSE10846 dataset were downloaded. Then, a LASSO regression analysis with ten-fold cross-validation recognized 36 of the 175 Bcl-2RGs as candidate gene sets for the prognostic signature. Multifactor Cox regression was used to identify the risk model containing Bcl-2RGs which was used to develop the Bcl-2RG signature. The risk score for patients in the cohort was calculated by the formula: $\text{Risk score }=\sum \begin{array}{l} N\\ i=1 \end{array}=(\exp\times coef)$, where N is the number of model genes, exp represents the gene expression value of each gene, and *coef* represents the coefficient index. The ‘time ROC’ package was applied to draw the time-dependent receiver operating characteristic (ROC) curve and determined the area under the curve (AUC) of the 2-, 3-, and 5-year overall survival (OS) rates. The risk scores of the GSE31312 and GES87371 datasets were computed and classified into high and low groups to validate the prognostic model using Kaplan–Meier survival analysis.

### 2.3 WGCNA to identify Bcl-2RGs

The ‘WGCNA’ package was used to identify Bcl-2 inhibitor resistance gene clusters based on the GSE10846 dataset. The weighted adjacency matrix was converted into a topological overlap matrix according to the optimal soft threshold (β=10), and then hierarchical clustering analysis was performed to detect the correlation between gene modules (minmodulesize=30; mergecutheight=0.25). Interaction strength was assessed using the heatmap toolkit, and gene significance and module membership were calculated to assess the relationship between the module and resistance characteristics.

### 2.4 Cell culture, viability, proliferation, and apoptosis

SU-DHL-8, SU-DHL-4, OCI-Ly3 and OCI-Ly10 cell lines were procured from Shanghai Zhong Qiao Xin Zhou Biotechnology Co., Ltd. Cells were maintained in Roswell Park Memorial Institute 1640 (RPMI) medium (Gibco, Waltham, MA, USA) supplemented with 10% fetal bovine serum (Gibco). Cell cultures were incubated at 37 °C in a humidified atmosphere containing 5% CO_2_, following the standard protocols provided by the supplier. Cell viability was detected using a Cell Counting Kit-8 (CCK-8; Protein Bio, Nanjing, China) according to the manufacturer’s protocol. Optical density was measured at 450 nm using a full-spectrum microplate reader. Cell proliferation was analyzed using a BeyoClick™ EdU Cell Proliferation Kit with Alexa Fluor 594 (C0078L, Beyotime Biotechnology) according to the manufacturer’s protocol. Apoptosis was evaluated using an Annexin V-FITC Apoptosis Detection Kit (C1062L, Beyotime Biotechnology) as previously described (11).

### 2.5 Western blotting

Cellular proteins were extracted and homogenized using RIPA lysis buffer (NCM, Suzhou, China). Protein samples were separated using SDS-PAGE (Epizyme, Shanghai, China) and transferred onto polyvinylidene fluoride membranes. Membranes were subsequently incubated with 5% skim milk to block non-specific binding sites, treated with primary and secondary antibodies, and protein bands were visualized using enhanced chemiluminescence reagents (NCM, Suzhou, China) and detected using an imaging system.

### 2.6 Statistical analysis

Data analysis, including basic statistical comparisons and IC50 determination was performed using GraphPad Prism v9.5 (GraphPad Software, CA, USA). Flow cytometry data were analyzed using FlowJo v10.8.1 (Tree Star, OR, USA). Bioinformatics analyses, including LASSO regression analysis, Multifactor Cox regression, Kaplan–Meier survival analysis, WGCNA analysis, C-index and DCA analysis were performed using R software v4.4.1 with the limma v3.60.4, survival v3.6-4, WGCNA v1.73, survcomp v1.54.0, ggplot2 v4.0.1, ggDCA v1.2 package. Western blot images were quantified using ImageJ software (NIH). GEO2R were used for Screening for DEGs, and jvenn for Venn diagram drawing. Statistical significance was set at P< 0.05. significant.

## 3 Results

### 3.1 Identification, establishment, and validation of Bcl-2RG prognostic signature

**Figure 1:**
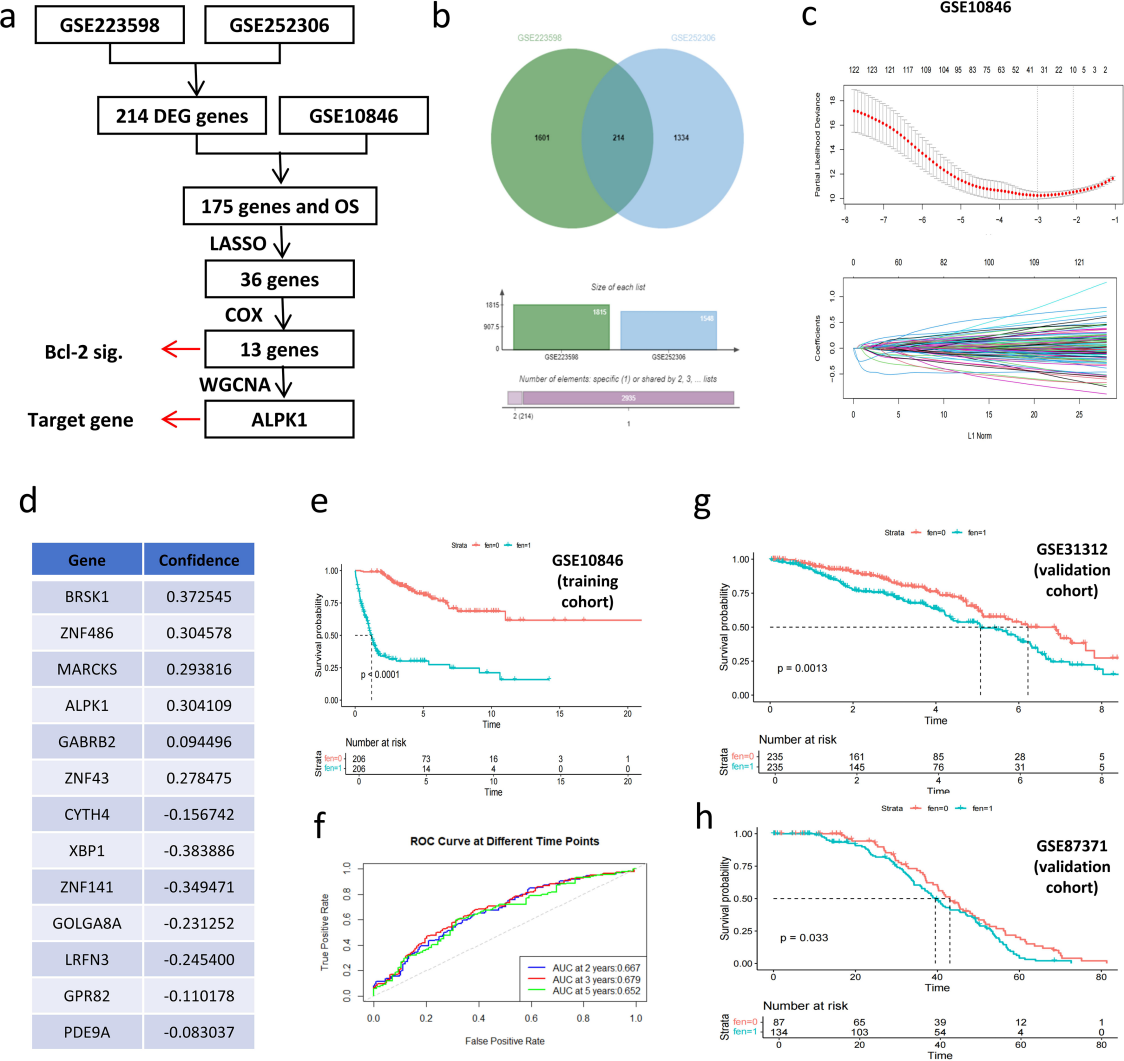
Identification, establishment, and validation of the Bcl-2RG prognostic signature. **(a)** Bcl-2RG prognostic signature identification process. **(b)** Venn diagram demonstrating that GSE223598 and GSE252306 shared 214 differentially expressed genes. **(c)** LASSO regression analysis of GSE10846. **(d)** The thirteen genes of the Bcl-2RG signature. **(e)** Kaplan–Meier survival curve of the high- and low-score groups in the training cohort (fen 0= low, fen 1= high). **(f)** Receiver operating characteristic curve of survival prediction in the training cohort (GSE10846). **(g, h)** Kaplan–Meier survival curves of the high- and low-score groups in the validation cohort (fen 0 = low, fen 1 = high). Bcl-2RG, Bcl-2 inhibitor-resistant gene.

The differentially expressed gene data from parental and venetoclax-resistant clonal cell lines (GSE223598 and GSE252306) were downloaded and analyzed to identify 214 Bcl-2RGs in DLBCL (**Figures 1a, b**). To clarify the relationship between Bcl-2RGs and the prognosis of DLBCL, 175 recorded Bcl-2RGs in the GSE10486 dataset (training cohort) and the OS of patients were analyzed. LASSO regression and ten-fold cross-validation identified 36 Bcl-2RGs as candidate gene sets for the prognostic signature (**Figure 1c**). Multifactor Cox regression results identified 13 Bcl-2RGs that were used to construct the prognostic model; their regression coefficients are shown in **Figure 1d**. The resistance score was obtained based on 13 Bcl-2RGs.The resistance score was estimated as follows:

Resistance Score = (0.372545 × *BRSK1*) + (0.304578 × *ZNF486*) + (0.293816 * *MARCKS*) + (0.304109 * *ALPK1*) + (0.094496 * *GABRB2*) + (0.278475* *ZNF43*) + (-0.156742 * *CYTH4*) + (- 0.383886 * *XBP1*) + (-0.349471 * *ZNF141*) + (-0.231252 * *GOLGA8A*) + (-0.2454 * *LRFN3*) + (- 0.110178 * *GPR82*) + (-0.083037 * *PDE9A*).

Patients in the GSE10846 dataset were classified into high- and low-drug resistance groups according to the median drug resistance score. Kaplan–Meier survival analysis revealed that patients with a high score (fen=1) had poorer OS than those with a low score (fen=0; **Figure 1e**). The AUCs of the 2-, 3-, and 5-year ROC curves were 0.667, 0.679, and 0.652, respectively, suggesting that the Bcl-2RG signature was highly useful for predicting outcomes (**Figure 1f**). Kaplan–Meier survival analysis showed that patients with high scores had shorter OS in the validation cohorts (GSE31312 and GSE87371; **Figures 1g, h**).

### 3.2 Inhibition of ALPK1 promotes DLBCL cell apoptosis and decreases proliferation

**Figure 2:**
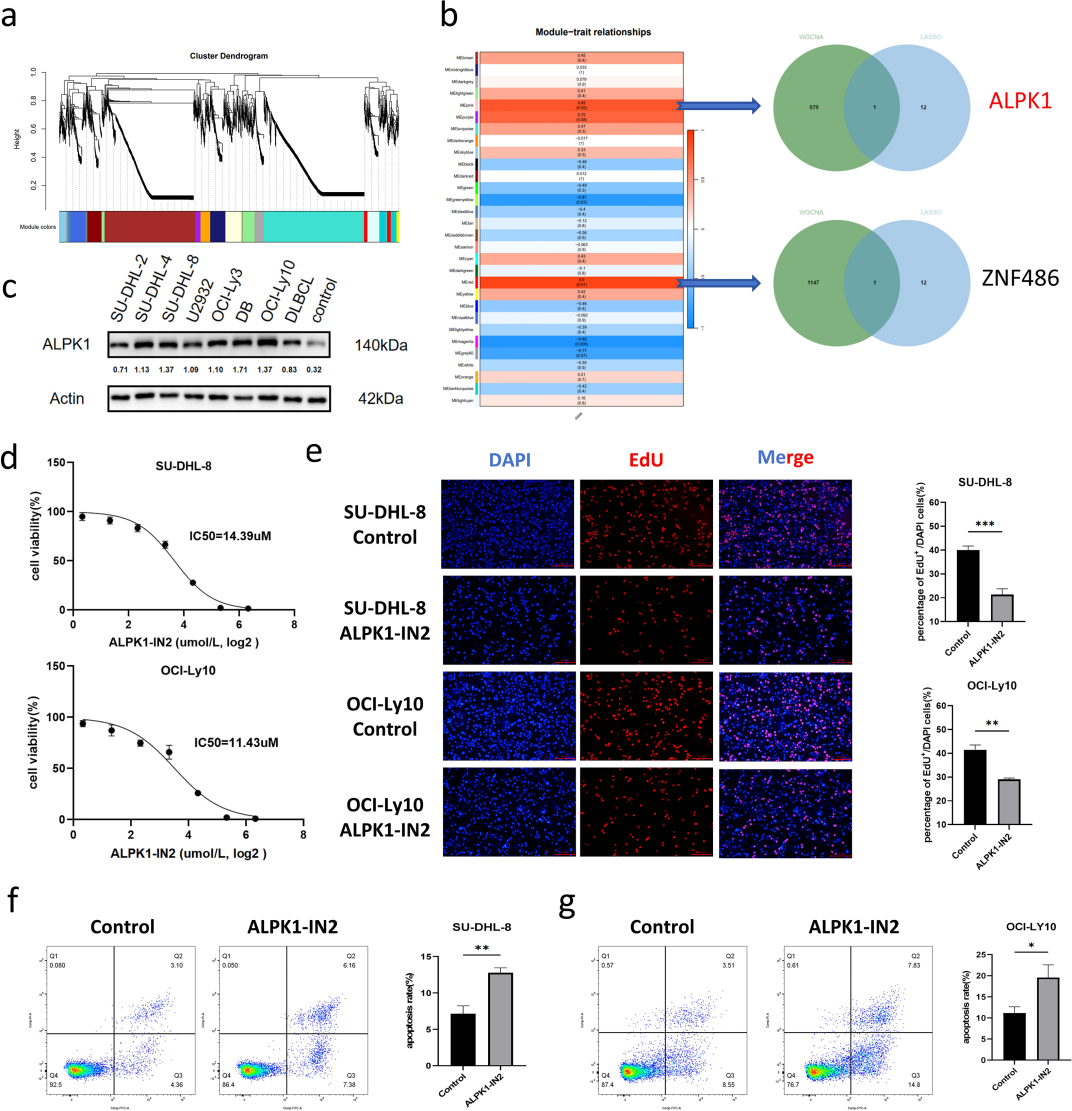
Inhibition of ALPK1 promotes DLBCL cell apoptosis and decreases proliferation. **(a)** Gene expression data were categorized into modules. **(b)** Association between the modules and clinical traits (left), and Venn diagram to identify overlapping genes from WGCNA and LASSO regression (right). **(c)** ALPK1 expression in different DLBCL cell lines (SU-DHL-2, SU-DHL-4, SU-DHL-8, U2932, OCI-LY3, DB, OCI-Ly10), peripheral blood mononuclear cells (DLBCL) from patients with DLBCL, and healthy donors (Control). **(d)** SU-DHL-8 and OCI-Ly10 cells were treated with the ALPK1 inhibitor ALPK1-IN2 for 24 **h** Cell viability was determined using the CCK8 assay. SU-DHL-8 were treated with 15 µM ALPK1-IN2 and OCI-Ly10 were treated with 10 µM ALPK1-IN2 for 24 hours. **(e)** Cell proliferation capacity was tested using EdU incorporation in different groups. The quantification shows the percentage of EdU-positive cells relative to total DAPI-stained cells (n=3 independent experiments). **(f, g)** Apoptosis detected by flow cytometry. Data are expressed as mean ± SD (*p<0.05, **p<0.01, ***<0.001). ALPK1, alpha protein kinase 1; DLBCL, diffuse large B-cell lymphoma.

To further clarify the biological function of Bcl-2RGs in DLBCL, the WGCNA algorithm was used to identify hub genes associated with resistance (**Figures 1a, b**). Thirty gene modules were identified, of which ME red and ME pink were strongly associated with Bcl-2 inhibitor resistance (**Figures 2a, b**). Functional enrichment analysis revealed that genes in this module are primarily involved in B cell receptor signaling pathway, apoptosis, and cell cycle (**Supplementary Figure S2**), highlighting a network-level shift towards B cell growth and apoptosis. Although these modules contain several highly connected Hub genes (such as KRAS and BCL2), we focus on ALPK1 because it is not only strongly associated with Venetoclax resistance, but also serves as a key prognostic factor in the Lasso-Cox model. A Venn diagram was used to identify overlapping genes (*ALPK1* and *ZNF486*) using WGCNA and LASSO analyses (**Figure 2b**). The ALPK1/NFκB axis plays an important role in innate immunity; however, less was known about the ALPK1/NFκB axis in DLBCL. We conducted experiments to verify the role of ALPK1 in the development and drug resistance of DLBCL. ALPK1 expression in DLBCL cell lines and patients with DLBCL was higher than that in healthy donors (controls; **Figure 2c**). The ALPK1 inhibitor, ALPK1-IN2, reduced SU-DHL-8 and OCI-Ly10 cell viability with IC50s of 11.39 and 11.43 uM, respectively (**Figure 2d**). Cell proliferation in SU-DHL-8 and OCI-Ly10 cells was evaluated by EdU incorporation assays, revealing a significantly lower percentage of EdU^+^ cells in the ALPK1-IN2 treated cells relative to untreated controls (**Figure 2e**; P< 0.05). DLBCL cell apoptosis increased in the ALPK1-IN2 treatment group compared to that in the control group (**Figures 2f, g**; P< 0.05).

### 3.3 Targeting ALPK1 enhances Bcl-2 inhibitor sensitivity by suppressing NFκB

**Figure 3:**
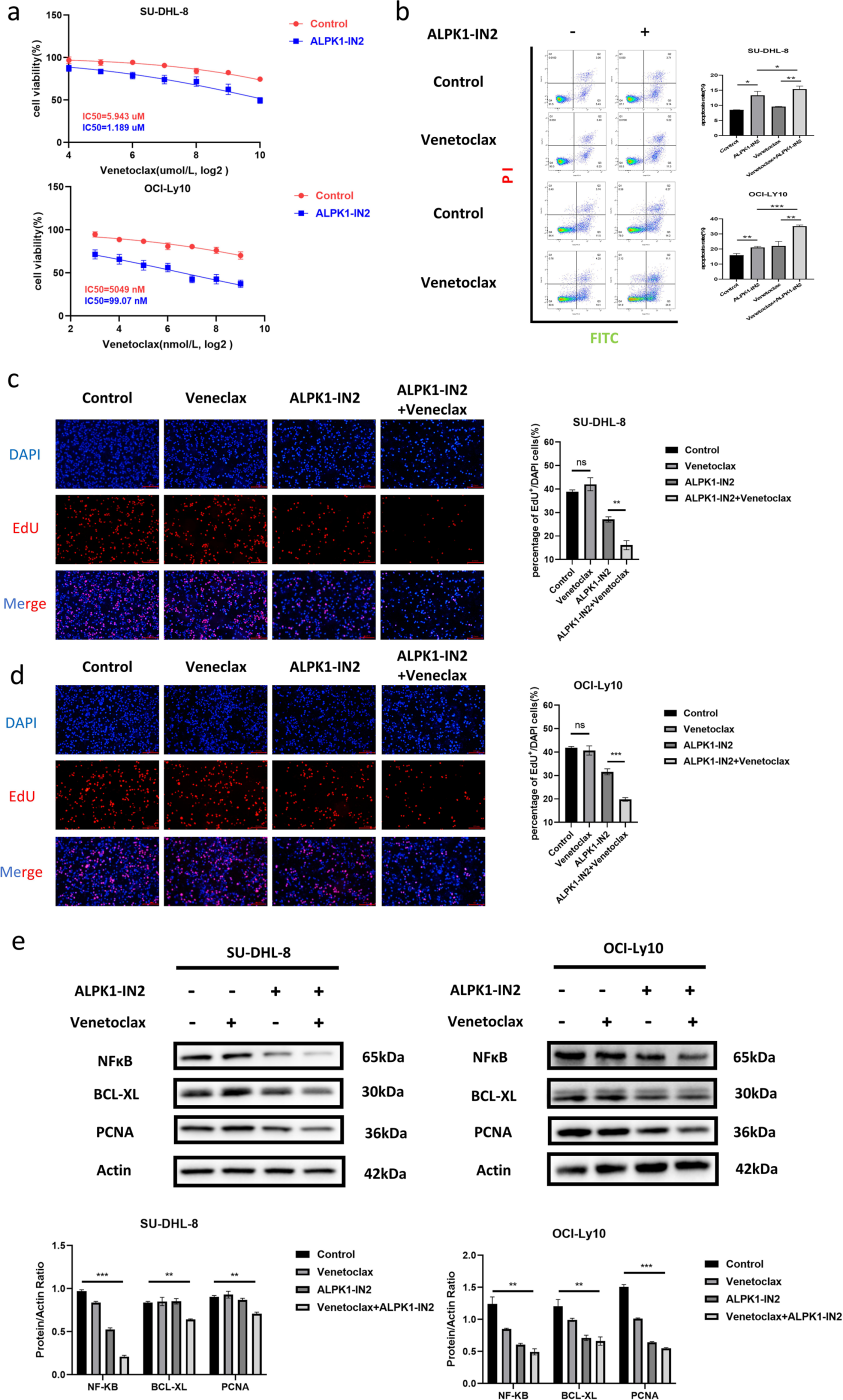
Targeting ALPK1 enhances Bcl-2 inhibitor sensitivity by suppressing NFκB and PCNA. **(a)** SU-DHL-8 and OCI-Ly10 cells were treated with DMSO plus increasing concentrations of venetoclax or the ALPK1 inhibitor ALPK1-IN2 plus venetoclax for 24 h (ALPK1-IN2 concentration: 15uM for SU-DHL-8 and 10uM for OCI-Ly10). Cell viability was determined using the CCK8 assay. SU-DHL-8 were treated with 15 µM ALPK1-IN2 and/or 1 µM venetoclax, OCI-Ly10 were treated with 10 µM ALPK1-IN2 and/or 100nM venetoclax for 24 h. **(b)** SU-DHL-8 and OCI-Ly10 cells were treated with DMSO plus venetoclax or ALPK1-IN2 plus venetoclax for 24 h. Apoptosis was detected using flow cytometry. **(c, d)** Cell proliferation capacity was tested using EdU incorporation in different groups. The quantification shows the percentage of EdU-positive cells relative to total DAPI-stained cells (n=3 independent experiments). **(e)** NFκB, BCL-XL, and PCNA expression of SU-DHL-8 and OCI-Ly10 cells treated with venetoclax, ALPK1-IN2, and a combination of venetoclax and ALPK1-IN2 were detected by western blotting. Band intensities were quantified using ImageJ and normalized to actin. Data are presented as mean ± SD (*p<0.05, **p<0.01, ***<0.001). ALPK1, alpha protein kinase 1; NFκB, nuclear factor kappa B; PCNA, proliferating cell nuclear antigen.

To determine whether ALPK1 is involved in venetoclax resistance in DLBCL and its mechanism of action, inhibitors of ALPK1 and Bcl-2 were used to treat DLBCL cell lines (SU-DHL-8 and OCI-Ly10). Cell viability was lower in the ALPK1-IN2-treated group than the control group The IC50s of venetoclax was reduced from 5.943 uM to 1.189 uM in SU-DHL-8 cells and from 5049 nM to 99.07 nM in OCI-Ly10 cells,(**Figure 3a**), indicating that ALPK1-IN2 might reverse venetoclax resistance. Venetoclax treatment significantly increased apoptosis in the ALPK1-IN2 group compared to the control group (**Figure 3b**). The percentage of EdU^+^ cells did not change after treatment with venetoclax in SU-DHL-8 or OCI-Ly10 cells. Combined treatment with ALPK1-IN2 reduced the percentage of EdU^+^ cells compared to control group and venetoclax treatment alone (**Figure 3c, d**).

ALPK1 is a protein kinase that plays an important role in activating the innate immune response to bacteria via NFκB signaling. NFκB is a hub gene in the BCR signaling pathway, which promotes the occurrence and progression of B-cell lymphoma by promoting proliferation and inhibiting apoptosis. Therefore, we hypothesize that ALPK1 might sensitize DLBCL to venetoclax by regulating NFκB signaling. As hypothesized, the expression of NFκB and its downstream signaling protein, BCL-XL, were reduced in the ALPK1-IN2 group compared to the control group, and significantly lower in the ALPK1-IN2 plus venetoclax group (P< 0.05; **Figure 3e**). The proliferation-associated protein PCNA was significantly downregulated in the ALPK1-IN2 plus venetoclax group compared to the other groups (P< 0.05; **Figure 3e**).

## 4 Discussion

Drug resistance leads to a poor prognosis in approximately 30–40% of patients with DLBCL undergoing first-line treatment following the R-CHOP regime. This drug resistance is accompanied by complex genetic changes and evolution. Identifying abnormally regulated genes related to drug resistance may provide new targets for predicting the prognosis of patients with DLBCL and for reversing drug resistance. Venetoclax, a Bcl-2 inhibitor approved by the FDA in 2017, brought about revolutionary breakthroughs in the treatment of CLL and AML. Recently, several clinical trials confirmed the role of venetoclax in improving the outcomes of patients with DLBCL. However, venetoclax resistance remains a major challenge in the treatment of patients with DLBCL. Therefore, it is necessary to explore the impact of Bcl-2RGs on drug sensitivity and prognosis to identify novel therapeutic targets, predict DLBCL prognosis, and to overcome venetoclax resistance. In this study, we uncovered a Bcl-2RGs signature that could predict the prognosis, and demonstrated that ALPK1 could be a therapeutic target for sensitizing DLBCL to venetoclax.

To elucidate the biological plausibility of our risk signature(Bcl-2RGs signature), we examined the potential roles of the constituent genes in the context of Venetoclax resistance and B-cell lymphoma biology. First, several genes are mechanistically linked to pathways known to confer resistance. ALPK1 is an upstream kinase that activates the NF-κB signaling pathway via the TIFA-TRAF6 axis (9). Given that constitutive NF-κB activity drives the expression of BCL-XL and MCL-1 in activated B-cell (ABC) DLBCL (12), ALPK1 likely functions as a novel upstream regulator of this resistance phenotype. Similarly, MARCKS, a prominent substrate of Protein Kinase C (PKC) (13), may reflect hyperactive BCR/PKC signaling, which supports B-cell survival and drug resistance. And XBP1 (specifically the spliced form, XBP1s) is a critical effector of the Unfolded Protein Response (UPR). Previous studies have demonstrated that XBP1s regulates MCL-1, a primary anti-apoptotic protein responsible for intrinsic resistance to BCL-2 inhibitors in DLBCL (14, 15). Second, our model identified several genes, including Zinc Finger proteins (ZNF486, ZNF141, and ZNF43) and metabolic regulators like PDE9A (16), which have not been previously reported in the context of venetoclax resistance. These genes likely represent novel transcriptomic features or potential biomarkers specific to the resistant cell state, suggesting new avenues for future mechanistic research.

ALPK1 is a cytosolic pattern-recognition receptor involved in promoting host cell defense through NFκB signaling, during bacterial infection (17). Recently, ALPK1 was shown to not only contribute to initial immunity but also to cancer progression. Lee et al. reported that ALPK1 regulates oral cancer progression (18), while Duizer et al. and Martin-Gallausiaux et al. demonstrated its role in to colorectal cancer(CRC) development (19, 20). Zhang et al. (9) showed that activating the ALPK1/NFκB signaling axis promoted extravasation and metastasis in CRC. NFκB being a crucial gene in the BCR signaling pathway, plays an important role in B cell lymphoma development. Moreover, NFκB signaling was chronically activated during the development of activated B-cell-like (ABC-) DLBCL (1). Thus, we hypothesized that the ALPK1/NFκB signaling axis may play an important role in DLBCL. Since this signaling axis is understudied, we focused on ALPK1 and not ZNF486 when choosing between the potential targets we identified (**Figure 2b**).

We conducted experiments to explore the impact of ALPK1 on DLBCL using its inhibitor, ALPK1-IN2. Following treatment with ALPK1-IN2, the proliferation of DLBCL cells decreased, and apoptosis increased (**Figures 2d–g**). Moreover, ALPK1-IN2 sensitized DLBCL cells to venetoclax (**Figures 3a–d**). These results suggest that ALPK1 may be a potential therapeutic target for reversing venetoclax resistance in DLBCL. Unexpectedly, venetoclax did not affect DLBCL cell proliferation, which was consistent with PCNA expression (**Figures 3c–e**). However, DLBCL cell proliferation and the level of PCNA expression significantly decreased when venetoclax was combined with ALPK1-IN2 compared to venetoclax treatment alone (**Figures 3c, d**). There have been no reports on the relationship between ALPK1 and PCNA and this interaction requires further study. In concert, the expressions of NFκB and BCL-XL were downregulated in cells treated with venetoclax plus ALPK1-IN2 (**Figure 3e**) compared to control cells, which correlated with increased apoptosis in combination-treated cells.

Ning et al. (21) suggested that the AGK-PTEN-FOXO1 signaling axis is involved in venetoclax resistance, while Portelinha et al. (22) showed that *SETD1B* mutations contribute to venetoclax resistance. In a study by Gomez Solsona et al. (23), metabolic reprogramming of glutamine was associated with venetoclax resistance. Although several studies have explored the mechanism of venetoclax resistance in DLBCL, no standard protocol for reversing venetoclax resistance has emerged. Therefore, the signature developed in our study by identifying Bcl-2RGs and therapeutic targets through the DEGs of different drug-resistant DLBCL cells (OCI-Ly1 and SU-DHL-16) combined with clinical prognostic information from multiple datasets (GES10846, GSE223598, and GSE252306) may provide a new avenue for the treatment of DLBCL.

Considering the influence of age and sex on genes, a multivariate Cox regression analysis was performed to adjust age and sex. The results showed that while the Risk Score maintained a hazard ratio (HR) > 1, its statistical significance was marginally affected (P>0.05) (**Supplementary Figure S3B**), likely due to the correlation between age-related gene expression and the risk signature. But the model integrating “Age + Sex + Risk Score” yielded a higher C-index (0.6414055) compared to the model with “Age + Sex” alone (0.6256885) (**Supplementary Figure S3C**). This indicates that the Risk Score provides incremental predictive value beyond standard clinical variables. Furthermore, the Decision Curve Analysis (DCA) demonstrated that the model integrating the Risk Score (Red line) had a higher net benefit compared to the base model (Green line) across a wide range of threshold probabilities (**Supplementary Figure S3D**). This indicates that the Risk Score adds valuable clinical utility for patient management.

## 5 Conclusion

We analyzed the Bcl-2 resistance-associated genes and established a reliable prognostic gene signature in DLBCL. Additionally, ALPK1 was identified as a target to reverse venetoclax resistance through the ALPK1/NFκB signaling axis and was established as a crucial gene involved in DLBCL progression and Bcl-2 inhibitor resistance.

## Data Availability

The original contributions presented in the study are included in the article/**Supplementary Material**. Further inquiries can be directed to the corresponding authors.
