# Supplementary material for: Bcl-2 inhibitor resistance in diffuse large b-cell lymphoma: establishing a prognostic signature and targeting alpha protein kinase 1
Source: Front Oncol. 2026 Jan 28;16:1729158. doi: 10.3389/fonc.2026.1729158 (PMC12890663; doi:10.3389/fonc.2026.1729158)
Supplement: Supplementary file 1 [file DataSheet1.pdf]

Supplementary Material 1. Souce data of Figure 2c.

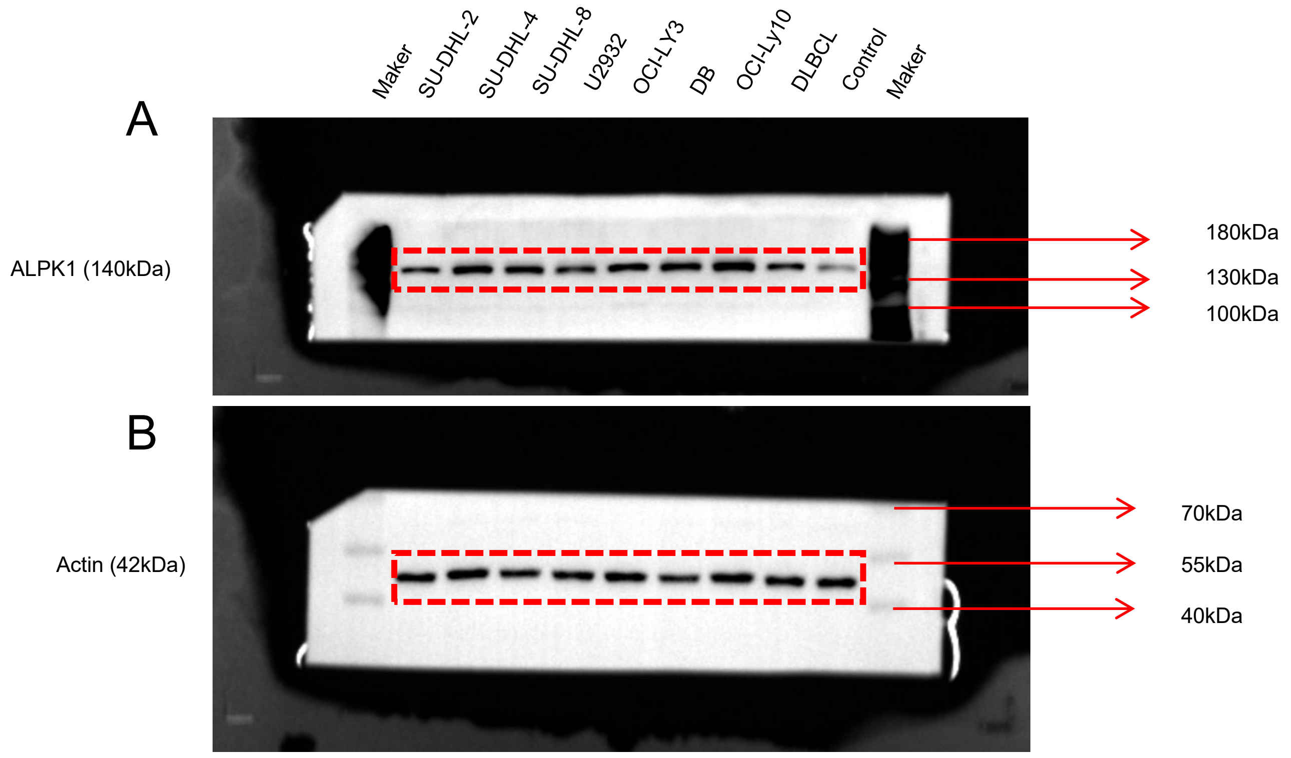

A, uncropped membrane of Figure 2c and Original blots image with Alpk1 polyclonal antibody.

B, uncropped membrane of Figure 2c and Original blots image with Actin polyclonal antibody.

Supplementary Material 2. Souce data of Figure 3e.

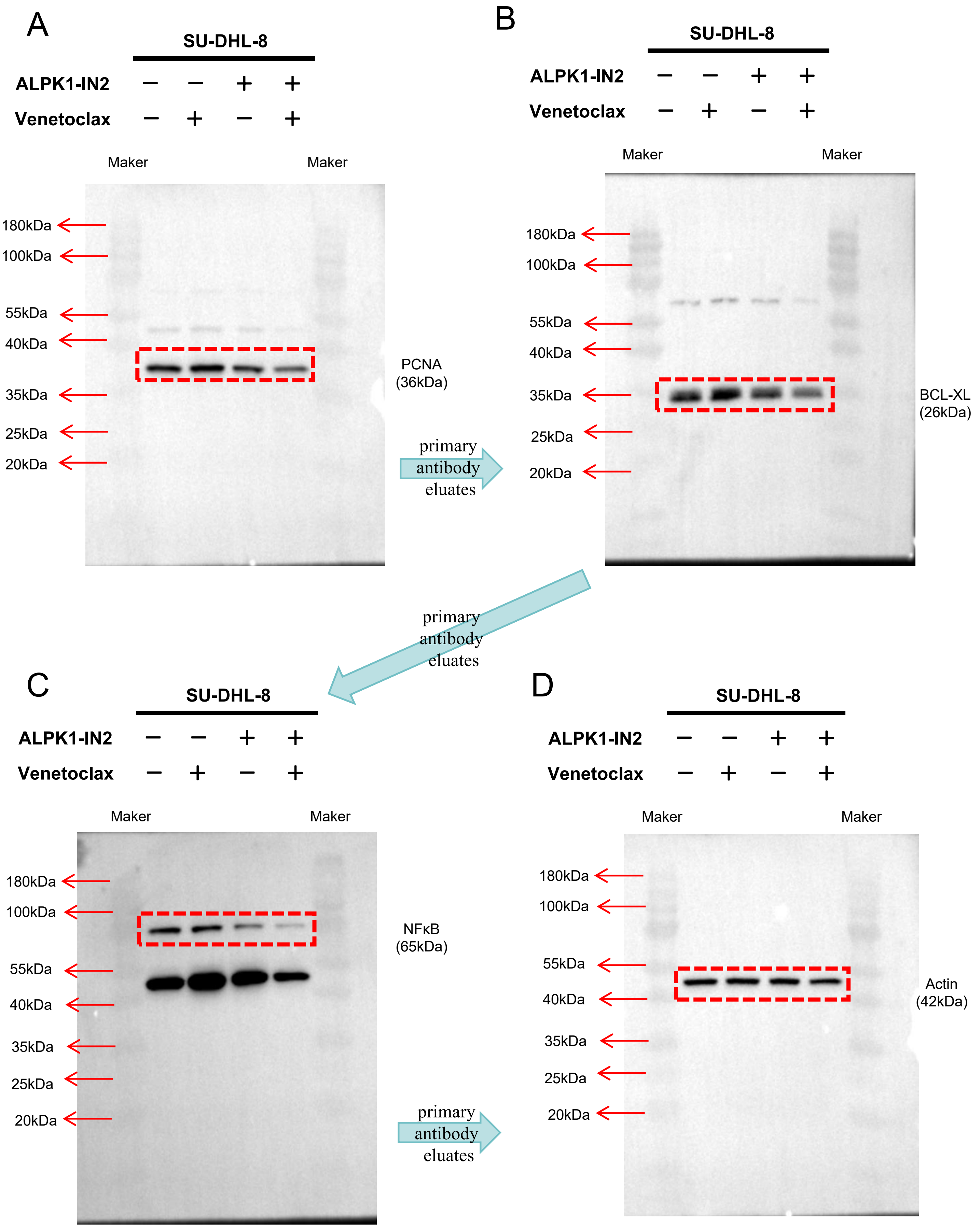

A, Full-length membrane of Figure 3e and Original blots image with PCNA polyclonal antibody.

B, Full-length membrane of Figure 3e and Original blots image with BCL-XL polyclonal antibody.

C, Full-length membrane of Figure 3e and Original blots image with NFκB polyclonal antibody.

D, Full-length membrane of Figure 3e and Original blots image with Actin polyclonal antibody.

Supplementary Material 3. Souce data of Figure 3e.

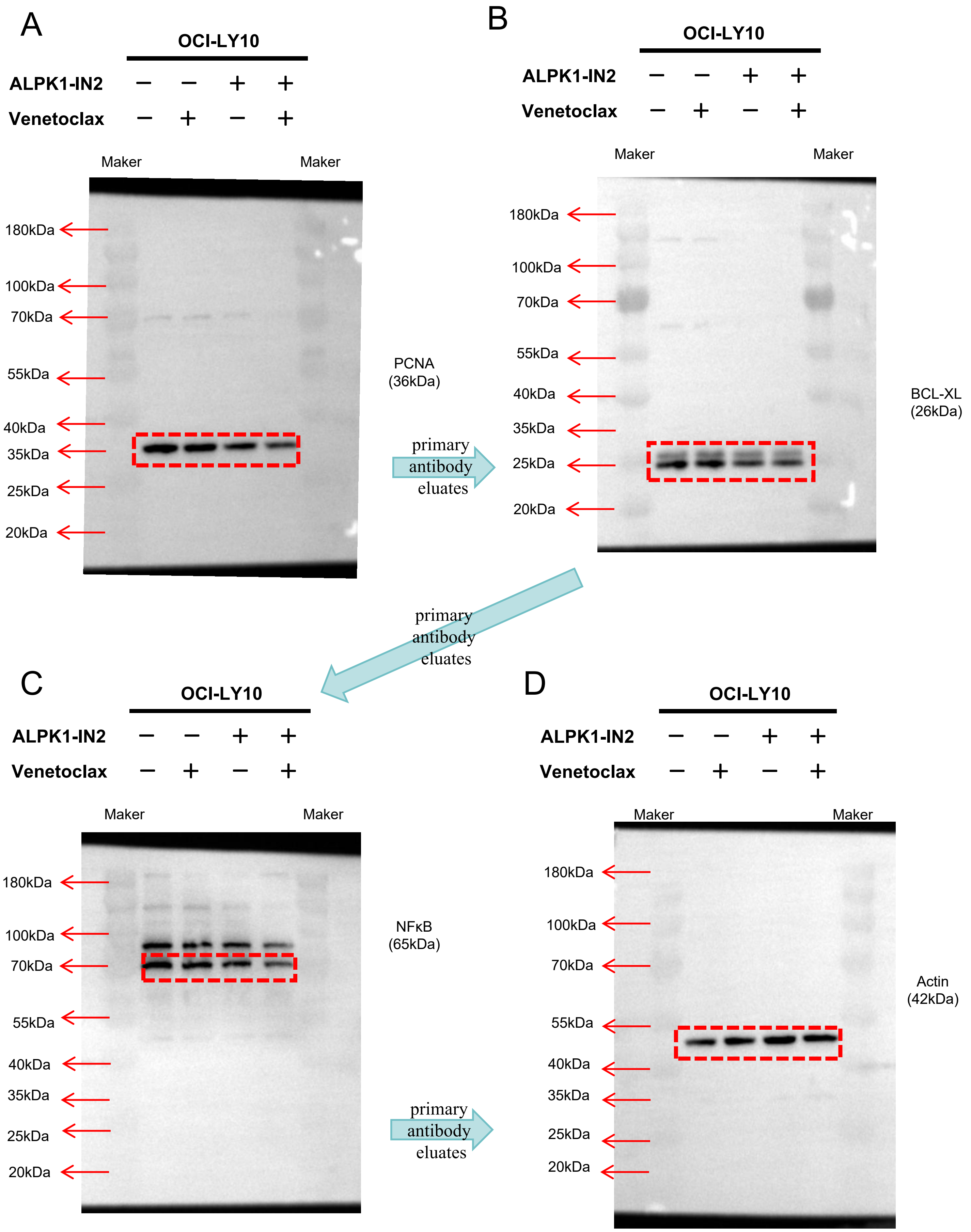

A, Full-length membrane of Figure 3e and Original blots image with PCNA polyclonal antibody.

B, Full-length membrane of Figure 3e and Original blots image with BCL-XL polyclonal antibody.

C, Full-length membrane of Figure 3e and Original blots image with NFκB polyclonal antibody.

D, Full-length membrane of Figure 3e and Original blots image with Actin polyclonal antibody.

Supplementary Material 4. Souce data of Figure 2e.

A

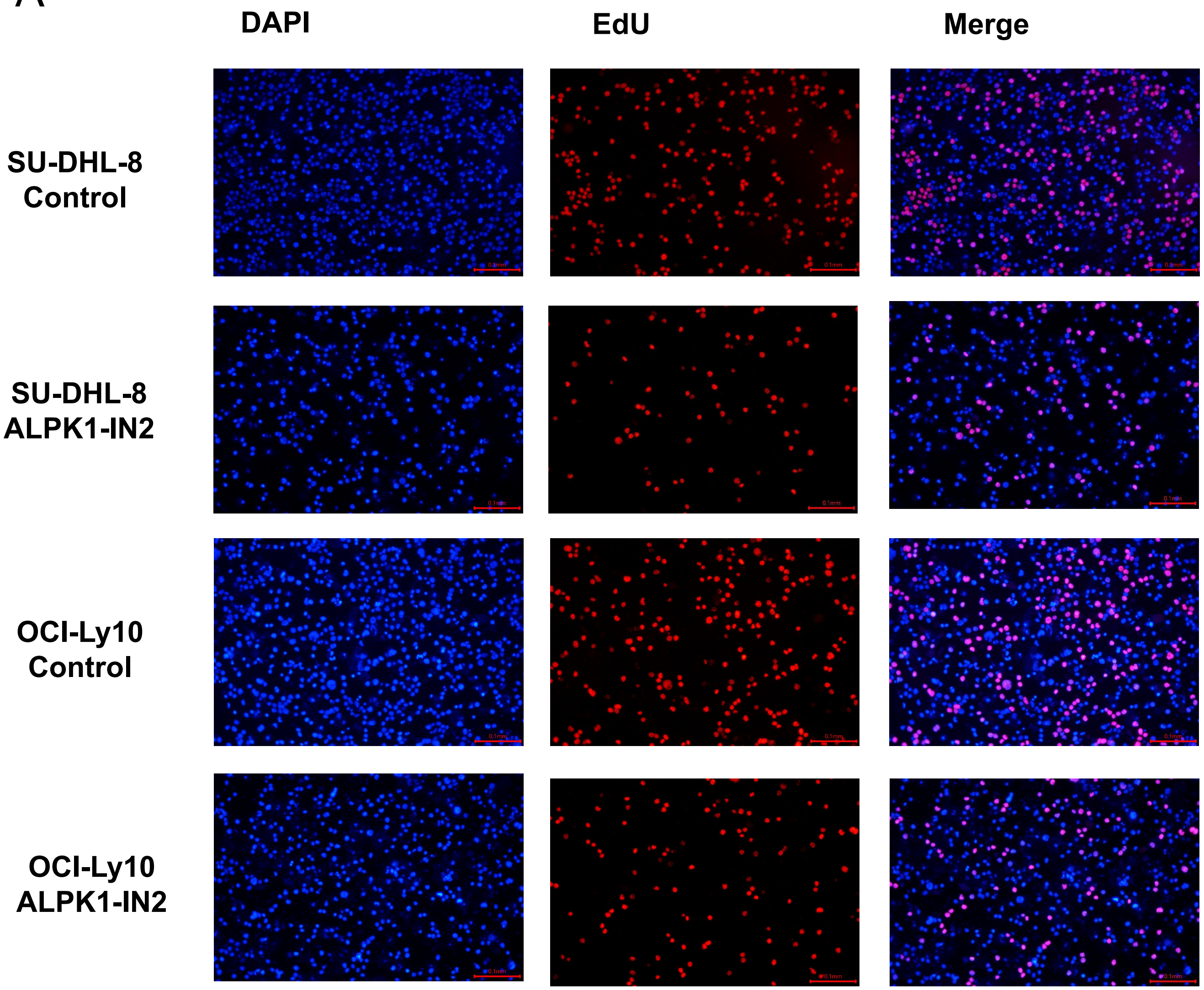

A, Uncropped, unedited parent images for all microscopic images of Figure 2e.

Supplementary Material 5. Souce data of Figure 3c and 3d.

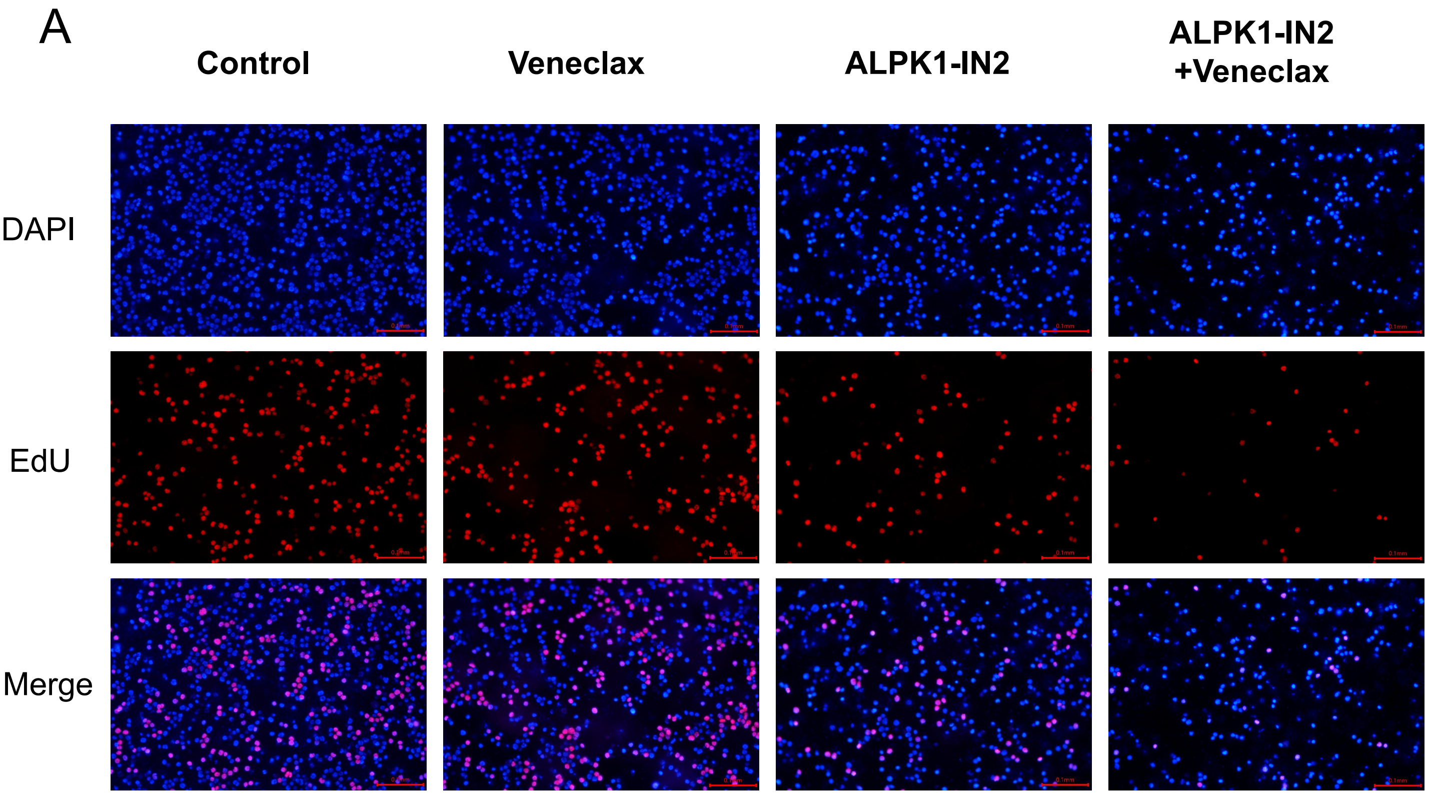

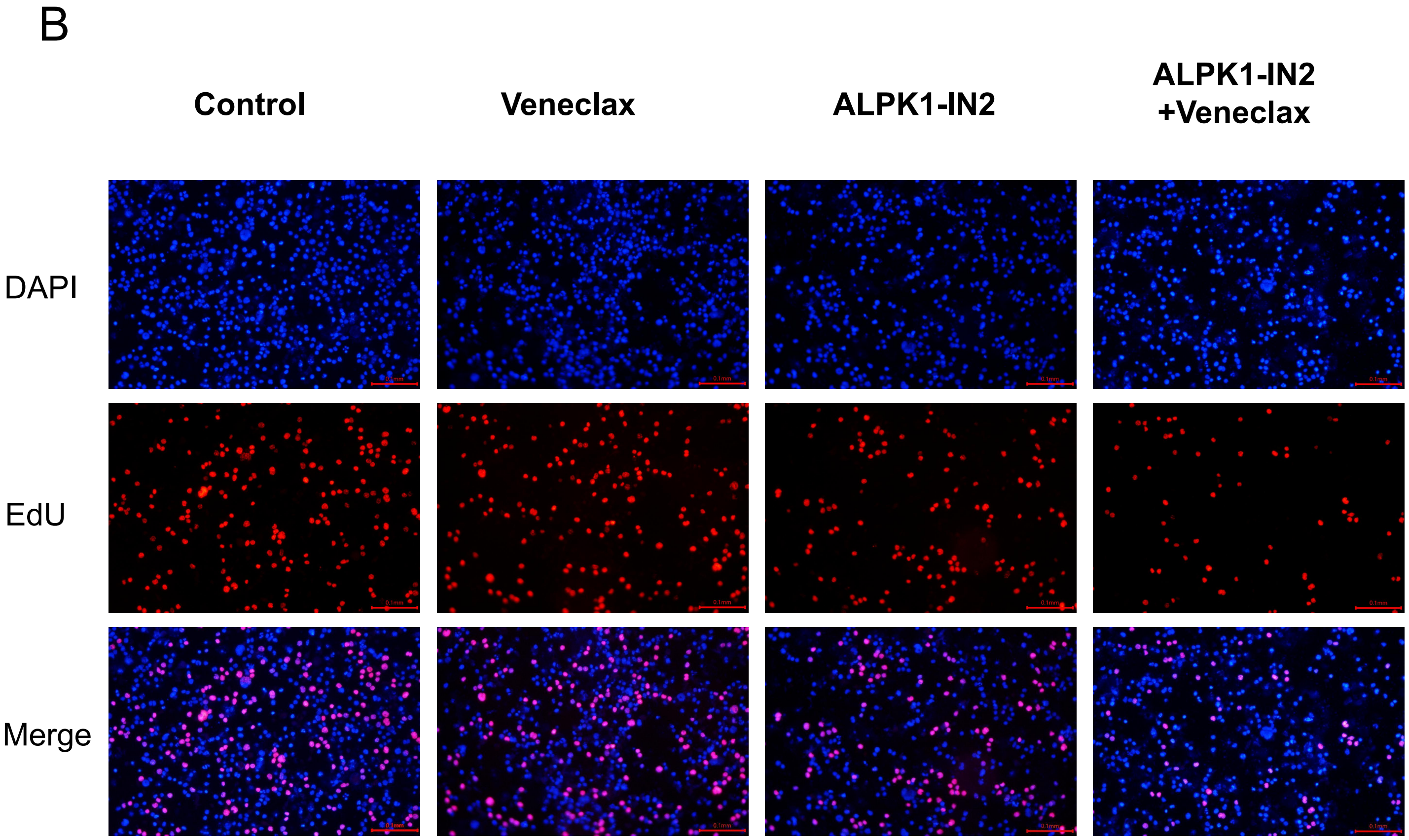

A, Uncropped, unedited parent images for all microscopic images of Figure 3c.

B, Uncropped, unedited parent images for all microscopic images of Figure 3d.
