# Supplementary figures and images for: Bcl-2 inhibitor resistance in diffuse large b-cell lymphoma: establishing a prognostic signature and targeting alpha protein kinase 1
Source: Front Oncol. 2026 Jan 28;16:1729158. doi: 10.3389/fonc.2026.1729158 (PMC12890663; doi:10.3389/fonc.2026.1729158)

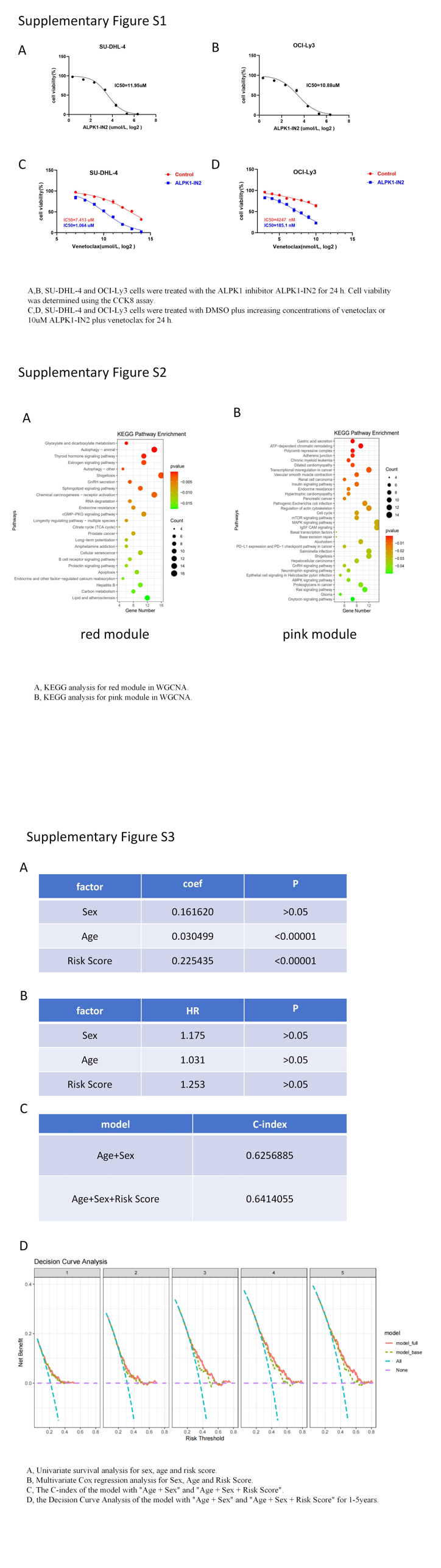

Supplement: Supplementary file 2 [file Image1.jpeg]
